# ZIP7 Drives Glycolytic Reprogramming and Lactate-Mediated Immune Remodeling in Lung Adenocarcinoma Through GSK3β-NRF2 Signaling

**DOI:** 10.3390/biomedicines14061262

**Published:** 2026-06-01

**Authors:** Zhihua Tang, Yueli Shi, Xinyuan Jiang, Sujing Jiang, Nueraili Maihemuti, Jie Zhang, Bufu Tang, Zhiyong Xu

**Affiliations:** 1 Institute of Drug Metabolism and Pharmaceutical Analysis, College of Pharmaceutical Sciences, Zhejiang University, Hangzhou 310058, China; 12319096@zju.edu.cn; 2 Department of Pharmacy, Shaoxing People’s Hospital (Shaoxing Hospital, Zhejiang University School of Medicine), Shaoxing 312000, China; 3 Zhejiang Cancer Hospital, Hangzhou Institute of Medicine (HIM), Chinese Academy of Sciences, Hangzhou 310022, China; 4 The Fourth Affiliated Hospital of School of Medicine, International School of Medicine, International Institutes of Medicine, Zhejiang University, Yiwu 322000, China; 5 Sir Run Run Shaw Hospital, School of Medicine, Zhejiang University, Hangzhou 310016, China; 6 Department of Interventional Radiology, Zhongshan Hospital, Fudan University, Shanghai 200032, China

**Keywords:** lung adenocarcinoma, ZIP7, glycolysis, NRF2, tumor-associated macrophages, anti-PD-1 therapy

## Abstract

**Background:** Zinc homeostasis regulated by ZIP transporters is critical for tumor glycolytic reprogramming and progression, yet the role of specific ZIP family members in lung adenocarcinoma (LUAD) remains unclear. This study aimed to identify the key ZIP transporter in LUAD and elucidate its molecular mechanisms and therapeutic value. **Methods:** siRNA-based functional screening of the ZIP family was performed in A549 and PC9 cells. A combination of in vitro cellular assays, in vivo animal models, clinical sample analysis and bioinformatics was used to validate the function of ZIP7 and explore its regulatory mechanisms. **Results:** ZIP7 (SLC39A7) was identified as a critical driver of glycolysis and proliferation in LUAD. It was significantly upregulated in LUAD tissues and cell lines. Mechanistically, ZIP7 increased inhibitory phosphorylation of GSK3β at Ser9 to stabilize NRF2, maintained low intracellular ROS levels, and sustained mTOR signaling to promote glycolytic flux. ZIP7-induced lactate secretion also drove M2-like macrophage polarization and PD-L1 upregulation to establish an immunosuppressive microenvironment. Notably, genetic or pharmacological inhibition of ZIP7 markedly enhanced the antitumor efficacy of anti-PD-1 therapy in vivo. **Conclusions:** ZIP7 is a pivotal oncogenic zinc transporter in LUAD that drives tumor progression via metabolic reprogramming and immune remodeling. Targeting ZIP7 represents a promising strategy to improve the efficacy of anti-PD-1 immunotherapy for LUAD.

## 1. Introduction

Essential metal elements are indispensable for maintaining human physiological functions, participating in nearly all biological processes through activation of signaling pathways, catalysis of enzymatic reactions, mediation of molecular transport, and stabilization of protein structures [1,2,3,4]. Among them, zinc ions (Zn^2+^) have attracted increasing attention. As the second most abundant trace element, Zn^2+^ acts not only as a structural cofactor and functional regulator for more than 3000 proteins but also as a dynamic “second messenger” that modulates intracellular signaling through “zinc waves”, thereby serving as a central determinant of cellular homeostasis [5,6].

A defining hallmark of cancer is metabolic reprogramming, wherein tumor cells preferentially rely on aerobic glycolysis (the Warburg effect) to meet their high biosynthetic and bioenergetic demands even in the presence of sufficient oxygen [7]. Beyond fueling rapid tumor growth, glycolytic reprogramming profoundly reshapes the tumor microenvironment (TME) through the accumulation of metabolic byproducts, with lactate being the most well-characterized. Elevated extracellular lactate levels exert multifaceted immunosuppressive effects: they directly impair the cytotoxic function and cytokine production of CD8^+^ T cells [8]; promote the polarization of tumor-associated macrophages (TAMs) toward an immune-suppressive M2 phenotype [9]; and drive the expansion and activation of myeloid-derived suppressor cells [10]. Collectively, these effects establish an immunosuppressive TME that facilitates tumor immune evasion and progression.

Increasing evidence shows that Zn^2+^ imbalance is tightly linked to this metabolic switch. Biochemically, Zn^2+^ inhibits glycolysis, for instance, by suppressing the activity of GAPDH, a critical glycolysis enzyme [11]. At the cellular and pathological level, Zn^2+^ imbalance broadly affects cell function and fate through glycolysis modulation. For example, in macrophages, Zn^2+^ enhances glycolysis and immune responses by inhibiting PP2A and activating the mTORC1-S6K pathway [12]. In neuronal cells, Zn^2+^ accumulation suppresses glycolysis, leading to energy failure and ultimately cell death [11]. In esophageal squamous cell carcinoma, Zn^2+^ deficiency enhances glycolysis and ferroptosis resistance [13], while clearance of free Zn^2+^ in cells inhibits glycolysis, alleviating benign prostatic hyperplasia [14]. These findings collectively suggest that Zn^2+^ may regulate tumor progression and immune evasion by modulating glycolytic metabolism, yet the precise mechanisms underlying this regulation in specific cancer types remain poorly defined.

Cellular Zn^2+^ homeostasis is maintained by the SLC39A/ZIP (influx) and SLC30A/ZnT (efflux/sequestration) families [15]. Among ZIP members, ZIP7 is an ER-resident channel that releases Zn^2+^ from intracellular stores into the cytosol [16]. Dysregulation of ZIP7 has been implicated in multiple cancers: it drives tamoxifen resistance and growth factor signaling in breast cancer [17], promotes bone metastasis via the MAZ-MYBL2 axis in prostate cancer [18], enhances proliferation and survival through Akt/mTOR in gastric cancer [19], and serves as a druggable node in the Notch pathway. Recent studies also link ZIP7 to ferroptosis regulation by modulating ER stress responses [20]. Despite these advances, whether and how ZIP7 controls glycolytic reprogramming and subsequent immunosuppression in cancers remain completely unresolved.

Lung cancer has the highest incidence and mortality rates globally. Lung adenocarcinoma (LUAD), as the main histological subtype, accounts for about 40–50% of all cases [21,22]. Given its clinical significance and the challenges in its treatment, the present study focused on the role of the ZIP zinc transporter protein family in LUAD’s metabolic reprogramming and malignant progression.

Here, through siRNA screening, we identified ZIP7 as a key regulator in LUAD. ZIP7 silencing suppressed glycolysis and proliferation. Mechanistically, ZIP7 inhibited GSK3β to stabilize NRF2, reducing ROS, inactivating AMPK, and activating mTOR to drive glycolytic reprogramming, while also promoting lactate-induced M2 TAM polarization. Targeting ZIP7 represents a promising strategy to inhibit tumor growth and enhance immunotherapy.

## 2. Materials and Methods

### 2.1. Cell Lines and Culture Conditions

Human LUAD cell lines (PC9, A549, H1975, H1650, H23), mouse lung cancer cell line Lewis Lung Carcinoma (LLC), and human normal lung epithelial cells (BEAS-2B) were obtained from ATCC. All cell lines underwent STR profiling for authentication and monthly mycoplasma testing using the MycoAlert Kit (Lonza, LT07-318, Basel, Switzerland). Cells were cultured at 37 °C with 5% CO_2_ in RPMI-1640 (PC9, A549, H1975, H1650, H23, BEAS-2B) or DMEM (LLC), both supplemented with 10% FBS and 1% penicillin/streptomycin. Cells were passaged at 80–90% confluency using 0.25% trypsin-EDTA, and cryopreserved in 90% FBS/10% DMSO freezing medium. The human monocytic cell line THP-1 was obtained from ATCC and maintained in RPMI-1640 with 10% FBS, 1% penicillin/streptomycin, and 0.05 mM β-mercaptoethanol. For macrophage differentiation, THP-1 cells were treated with 100 ng/mL PMA (Sigma-Aldrich, P8139, St. Louis, MO, USA) for 48 h. Differentiated THP-1-derived macrophages were validated by flow cytometric analysis of CD68 expression.

### 2.2. Genetic Manipulations

The siRNA library targeting the human ZIP zinc transporter protein family and negative control siRNA were purchased from Ribobio (Guangzhou, China). For the primary siRNA screen, A549 and PC9 cells were seeded in 12-well plates and transfected with a mixture of 3 independent siRNAs (at a 1:1:1 ratio) using Lipofectamine RNAiMAX (Invitrogen, 13778150, Carlsbad, CA, USA) at a final concentration of 45 nM. Forty-eight hours after transfection, the medium was replaced with fresh medium, and the cells were cultured for another 24 h. Lactate production was assessed using an assay kit (Sigma-Aldrich, MAK064, St. Louis, MO, USA), and cell viability was evaluated with the CCK-8 reagent. All results were normalized to those of cells transfected with negative control siRNA. The top candidate (ZIP7) was selected for follow-up validation using stable shRNA-mediated knockdown. To this end, lentiviral vectors encoding ZIP7-specific shRNA (sequences in Supplementary Table S1) or control shRNA were packaged in HEK293T cells using psPAX2/pMD2.G plasmids. Target cells were transduced at MOI = 5 with polybrene, followed by puromycin selection (2 μg/mL, 7–10 days). ZIP7 overexpression cell lines were generated by lentiviral transduction of full-length human ZIP7 cDNA cloned into pLVX-puro, with puromycin selection and validation by Western blot.

### 2.3. Conditioned Medium Preparation and Macrophage Polarization Induction

LUAD cells (control/ZIP7-KD/ZIP7-OE) were washed with PBS and incubated in serum-free RPMI-1640 for 24 h. Conditioned medium (CM) was centrifuged (2000× *g*, 10 min), filtered (0.22 μm), and stored at −80 °C. THP-1-derived macrophages were incubated with the collected CM in the presence or absence of 10 mM lactate, and M2 macrophage polarization was subsequently assessed.

### 2.4. Immunohistochemistry (IHC) and Immunofluorescence (IF)

Formalin-fixed paraffin-embedded tissue sections (4 μm) were processed for both IHC and IF staining. Firstly, the sections were subjected to deparaffinization treatment, and then antigen retrieval was carried out according to the recommendations of Cell Signaling Technology (Danvers, MA, USA). For IHC analysis targeting Ki67 and ZIP7, sections were incubated with primary antibodies at specified dilutions: Ki67 (1:200) and ZIP7 (1:200). Detection was performed using HRP-conjugated secondary antibodies, followed by DAB chromogen development and hematoxylin counterstaining. For IF staining, sections were first incubated with the CD206 primary antibody (1:100), and then Alexa Fluor 488 fluorescently conjugated secondary antibodies (Invitrogen, # A32731, Carlsbad, CA, USA) were used. IHC and IF images were captured using a light microscope and an Olympus FV3000 confocal microscope (Olympus, Tokyo, Japan), respectively. Finally, the IHC score, the quantity of Ki67 positive cells in IHC, and that of CD206 positive cells in IF were calculated using Image J 1.49v software.

### 2.5. Human Samples Collection

LUAD tumor and adjacent normal tissues were obtained from the Fourth Affiliated Hospital of Zhejiang University School of Medicine (Yiwu, China) and pathologically confirmed. All patients provided written informed consent. The study was approved by the Research Ethics Committee of the Fourth Affiliated Hospital of Zhejiang University School of Medicine (No. K2024211) and followed the Declaration of Helsinki.

### 2.6. Western Blot

Proteins were extracted with RIPA buffer containing protease/phosphatase inhibitors, quantified by BCA assay, separated via SDS-PAGE (6–12% gels), and transferred to PVDF membranes. Immunoblotting was performed with primary antibodies against ZIP7, AMPK/mTOR pathway components, NRF2, GSK3β/p-GSK3β, and ubiquitin, followed by HRP-conjugated secondaries and ECL detection. Antibody information is presented in Supplementary Table S2.

### 2.7. RNA Extraction and Quantitative PCR

Total RNA was isolated from cells or tissues using TRIzol reagent (Beyotime, R0016, Shanghai, China). After measuring purity/concentration (NanoDrop, Wilmington, DE, USA), RNA was reverse transcribed with the PrimeScript RT Kit (Takara, RR037A, Shiga, Japan). qPCR was performed using ChamQ Universal SYBR qPCR Master Mix (Vazyme, Q711-02, Nanjing, China) on a QuantStudio 6 system with gene-specific primers (Supplementary Table S3). Relative expression was calculated using the 2^−△△Ct^ method normalized to 18s rRNA.

### 2.8. ROS Detection

Intracellular ROS was measured by incubating cells with 10 μM DCFH-DA (Sigma-Aldrich, D6883, St. Louis, MO, USA) for 20 min followed by flow cytometry. Mitochondrial ROS was assessed by incubating cells with 5 μM MitoSOX Green (Invitrogen, M36006) for 30 min. Representative fluorescent images were captured using a confocal microscope (FV3000, Olympus), and fluorescence intensity scores were determined using Image J software.

### 2.9. Cell Proliferation Assays

For the CCK-8 assay, cells were seeded in 96-well plates, cultured overnight, and then incubated with 10 μL CCK-8 reagent (Beyotime, C0037) at 37 °C for 2 h. Absorbance at 450 nm was measured using a microplate reader. For the EdU assay, cells were incubated with 10 μM EdU (Beyotime, C0071S, Shanghai, China) at 37 °C for 1 h, fixed with 4% paraformaldehyde (15 min), permeabilized with 0.5% Triton X-100 (Beyotime Biotechnology, Shanghai, China) (20 min), and stained with Alexa Fluor 594-azide (Beyotime Biotechnology, Shanghai, China). Nuclei were counterstained with DAPI and imaged.

### 2.10. Apoptosis Assay

Cells were stained with Annexin V-FITC and PI using the corresponding kit according to the manufacturer’s instructions (Beyotime, C1062S). Apoptotic populations (Annexin V^+^/PI^−^ and Annexin V^+^/PI^+^) were quantified by flow cytometry.

### 2.11. Cell Migration and Invasion

For the wound healing assay, confluent monolayers were scratched with a pipette tip. Wound closure was calculated as [(initial scratch width − width at 48 h)/initial width] × 100% by measuring scratch widths at 0 and 48 h.

For the cell migration assay, transwell chambers (8-μm pore size) without Matrigel coating were used. Cells were suspended in serum-free medium and seeded into the upper chambers at a density of 3 × 10^4^ cells per well. The lower chambers were filled with complete medium containing 10% FBS as a chemoattractant. After 24 h of incubation, non-migrated cells on the upper side of the membrane were removed, and migrated cells on the lower side were fixed, stained with 0.1% crystal violet, and counted in five randomly selected fields under a light microscope.

For cell invasion assay, the same transwell chambers were pre-coated with 50 μL of Matrigel (diluted 1:8 in serum-free medium) and incubated at 37 °C for 6 h to solidify. Cells were then seeded at the same density (3 × 10^4^ cells per well) in serum-free medium into the upper chambers, with 10% FBS in the lower chambers as a chemoattractant. After 48 h of incubation, non-invading cells were removed, and invading cells on the lower membrane surface were fixed, stained with 0.1% crystal violet, and quantified using the same counting method as in the migration assay. All experiments were performed in triplicate.

### 2.12. Colony Formation Assay

Logarithmic phase cells were trypsinized, counted, and seeded into 6-well plates at 2000 cells/well. Cells were cultured in complete medium for 10–14 days (medium changed every 3–4 days). After visible colonies formed, cells were washed with PBS, fixed with 4% PFA for 15–20 min, and stained with 0.1% crystal violet for 20–30 min at room temperature. Excess stain was washed off with distilled water, and colonies with >50 cells were manually counted under a light microscope. Each experiment was performed in triplicate.

### 2.13. Glycolysis Assays

Glucose uptake was measured using 100 μM 2-NBDG (Thermo Fisher, N13195, Waltham, MA, USA), with fluorescent intensity analyzed by Image J software after imaging. Lactate production was quantified using an assay kit (Sigma-Aldrich, MAK064, St. Louis, MO, USA) according to the manufacturer’s instructions. The extracellular acidification rate (ECAR), a surrogate marker of glycolysis activity, was analyzed using the Seahorse XF Glycolysis Stress Test (Agilent Technologies, Santa Clara, CA, USA) on an XF96 Extracellular Flux Analyzer. ATP levels were determined using the ATP Assay Kit (Beyotime, S0026) following the manufacturer’s instructions.

### 2.14. In Vivo Tumor Models

For the subcutaneous xenograft model, LLC cells (1 × 10^6^ cells/mouse) suspended in 100 μL PBS were injected subcutaneously into the right flank of C57BL/6 mice. Tumor volume was monitored every 2 days (using the formula V = 0.5 × length × width^2^). When the tumors grew to approximately 100 mm^3^, treatments began. ZIP7 inhibitor NVS-ZP7-4 (ZP74, MCE, HY-114395) was administered intraperitoneally (i.p.) at doses of 5 mg/kg or 10 mg/kg every two days, and anti-PD-1 (200 µg anti-mouse) was also given i.p. every two days. Endpoints were set as tumor volume reaching or exceeding 2000 mm^3^ or the occurrence of ulceration. The experiment was conducted in line with relevant animal ethics regulations.

For the lung metastasis model, C57BL/6 mice received an injection of LLC cells (1 × 10^6^ cells/mouse) in 100 μL sterile PBS via the tail vein. ZP74 treatments (same as the subcutaneous model) started 24 h post-injection. Lungs were harvested on day 30 and fixed in 4% paraformaldehyde. Then, surface metastatic nodules were counted macroscopically, and the paraffin sections were prepared for further analysis. Mice used in all in vivo experiments in this study were randomly assigned to different treatment groups to ensure no significant differences in body weight and general condition among groups before treatment, thereby avoiding grouping bias. Blinding was strictly implemented throughout the experiment: investigators responsible for animal injection, tumor volume measurement, counting of lung metastatic nodules, and data analysis were unaware of group allocation, effectively eliminating subjective bias caused by human factors. All animal experiments were approved by the Animal Care and Use Committee of the Zhejiang University School of Medicine (Approval No. ZJU20250008).

### 2.15. Bioinformatics Analysis

To verify ZIP7 (SLC39A7) mRNA and protein expression in LUAD and normal tissues, GEPIA (http://gepia.cancer-pku.cn/, accessed on 7 March 2025) and UALCAN (https://ualcan.path.uab.edu/, accessed on 7 March 2025) databases were used.

### 2.16. Statistical Analysis

Statistical analyses were all carried out using GraphPad Prism 9.0, and data are presented as mean ± SD. Survival curves were analyzed by log-rank test. Statistical significance was determined by two-tailed Student’s *t*-test or one-way ANOVA with Tukey’s multiple-comparison test, as appropriate. Significance was defined as follows: * *p* < 0.05, ** *p* < 0.01, *** *p* < 0.001. The number of biological replicates was set as ≥3 experiments for in vitro studies and ≥5 mice per group for in vivo studies.

## 3. Results

### 3.1. Functional Screening Identifies ZIP7 as a Glycolysis-Linked Dependency in LUAD

**Figure 1:**
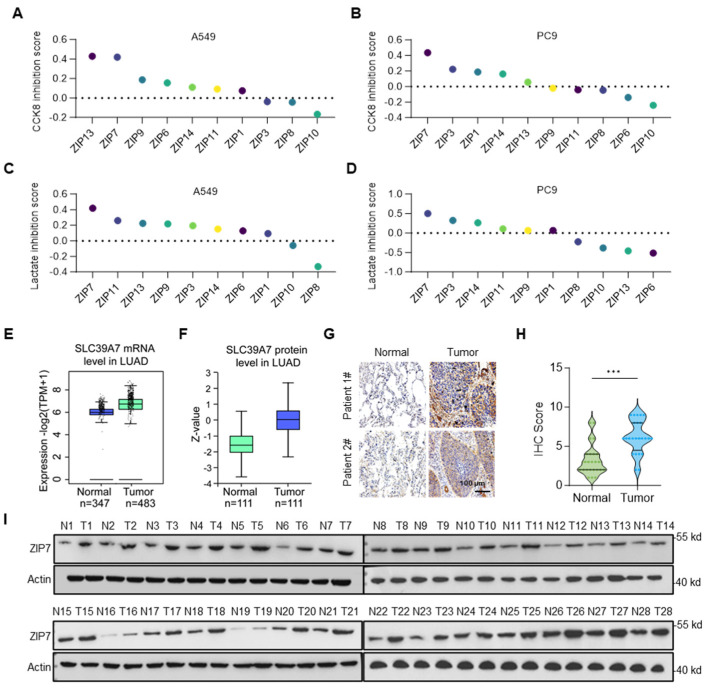
ZIP7 is identified as a glycolysis-associated dependency and is upregulated in LUAD. (**A**,**B**) CCK8 inhibition scores of individual ZIP family members in A549 and PC9 cells following transient silencing, ranked according to growth-inhibitory effects. (**C**,**D**) Lactate inhibition scores of individual ZIP family members in A549 and PC9 cells following transient silencing, ranked according to suppression of lactate production. (**E**) SLC39A7 mRNA levels in normal lung tissues and LUAD tissues were analyzed using the GEPIA database (http://gepia.cancer-pku.cn, accessed on 7 March 2025). (**F**) ZIP7 protein levels were analyzed in CPTAC samples by UALCAN (https://ualcan.path.uab.edu/analysis.html, accessed on 7 March 2025). (**G**) Representative immunohistochemical staining of ZIP7 in paired normal and LUAD tissues. Scale bar, 100 μm. (**H**) Quantification of ZIP7 IHC scores in normal and tumor tissues (*n* = 24), *** *p* < 0.001. (**I**) Immunoblot analysis of ZIP7 protein expression in paired normal (N) and tumor (T) lung tissues. Actin served as a loading control (*n* = 28). Data are presented as mean ± SD unless otherwise indicated. Statistical significance is indicated in the panels.

To identify ZIP transporters that couple glycolytic output to malignant fitness in LUAD, we performed a functional loss-of-function screen across the ZIP family in A549 and PC9 cells using cell viability and lactate output as parallel readouts (Figure S1A). Ranking analysis showed that ZIP7 was the only candidate that consistently scored among the top hits in both cell lines and across both phenotypes, nominating it as a glycolysis-associated dependency in LUAD (Figure 1A–D). We next assessed the clinical relevance of ZIP7 and found that SLC39A7/ZIP7 was broadly elevated across multiple tumor types and significantly upregulated in LUAD at both the mRNA and protein levels (Figure S1B and Figure 1E,F). ZIP7 mRNA expression was also higher in LUAD cell lines than in normal bronchial epithelial cells (Figure S1C). Importantly, paired clinical samples further confirmed increased ZIP7 expression in tumor tissues by IHC and immunoblotting (Figure 1G–I). These findings identify ZIP7 as a clinically relevant candidate linked to glycolytic fitness in LUAD.

### 3.2. ZIP7 Sustains Aerobic Glycolysis and Glycolytic ATP Production

**Figure 2:**
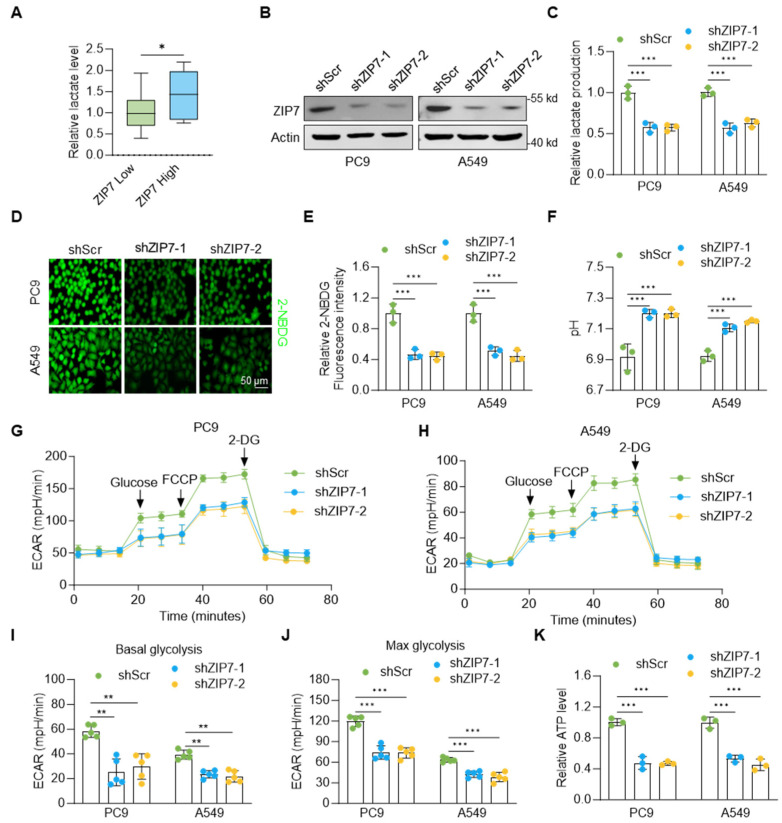
**ZIP7 sustains aerobic glycolysis in LUAD cells.** (**A**) Relative lactate levels in LUAD tissues stratified by low or high ZIP7 expression, * *p* < 0.05. (**B**) Immunoblot validation of ZIP7 knockdown efficiency in PC9 and A549 cells. Actin served as a loading control. (**C**) Relative lactate production in control and ZIP7-knockdown PC9 and A549 cells (*n* = 3), *** *p* < 0.001. (**D**) Representative images of 2-NBDG uptake in control and ZIP7-knockdown PC9 and A549 cells. Scale bar, 50 μm. (**E**) Quantification of relative 2-NBDG fluorescence intensity shown in (**D**), *n* = 3, *** *p* < 0.001. (**F**) Measurement of extracellular pH in culture medium from control and ZIP7-knockdown PC9 and A549 cells (*n* = 3), *** *p* < 0.001. (**G**,**H**) Extracellular acidification rate (ECAR) profiles of control and ZIP7-knockdown PC9 and A549 cells during Seahorse glycolysis stress testing. (**I**,**J**) Quantification of basal glycolysis and maximal glycolysis derived from the ECAR assays shown in (**G**,**H**), *n* = 5, ** *p* < 0.01, *** *p* < 0.001. (**K**) Relative ATP levels in control and ZIP7-knockdown PC9 and A549 cells (*n* = 3), *** *p* < 0.001. Data are presented as mean ± SD. Statistical significance is indicated in the panels.

Given the association between ZIP7 expression and lactate abundance in LUAD tissues (Figure 2A), we next investigated whether ZIP7 directly regulates glycolysis. Based on ZIP7 expression profiling across cell lines, we selected A549 and PC9 (high expression) for knockdown and H1975 (low expression) for overexpression. Stable ZIP7 knockdown in PC9 and A549 cells was confirmed by immunoblotting (Figure 2B). ZIP7 depletion significantly reduced lactate production and glucose uptake while increasing medium pH (Figure 2C–F). Seahorse analysis further showed that ZIP7 knockdown markedly suppressed ECAR in both PC9 and A549 cells, accompanied by decreases in basal glycolysis and maximal glycolytic capacity (Figure 2G–J). Consistently, ATP levels were also reduced upon ZIP7 silencing (Figure 2K). In contrast, ZIP7 overexpression in H1975 cells enhanced lactate production, glucose uptake, and glycolytic capacity while decreasing extracellular pH (Figure S2A–H). Together, these results demonstrate that ZIP7 is required to sustain aerobic glycolysis and glycolysis-associated ATP production in LUAD cells.

### 3.3. ZIP7 Contributes to LUAD Growth and Metastasis In Vitro and In Vivo

**Figure 3:**
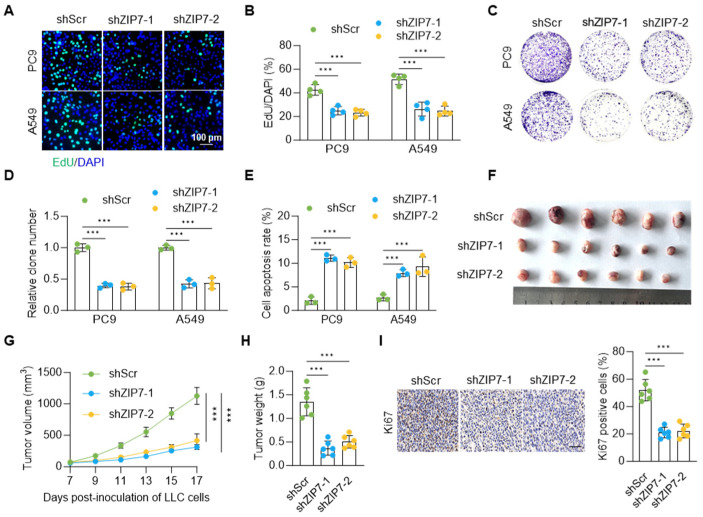
ZIP7 promotes LUAD cell proliferation and tumor growth. (**A**) Representative EdU staining images of control and ZIP7-knockdown PC9 and A549 cells. Scale bar, 100 μm. (**B**) Percentage of EdU-positive cells relative to total DAPI-stained nuclei (*n* = 4), *** *p* < 0.001. (**C**) Representative colony formation images of control and ZIP7-knockdown PC9 and A549 cells. (**D**) Colonies (>50 cells) were counted and normalized to shScr control (*n* = 3), *** *p* < 0.001. (**E**) Apoptosis rates in control and ZIP7-knockdown PC9 and A549 cells (*n* = 3), *** *p* < 0.001. (**F**) Representative images of subcutaneous tumors derived from LLC cells expressing control shRNA or shZIP7. (**G**) Tumor growth curves of the indicated groups in the LLC subcutaneous xenograft model (*n* = 6), *** *p* < 0.001. (**H**) Final tumor weights from the indicated groups (*n* = 6), *** *p* < 0.001. (**I**) Representative Ki67 immunohistochemical staining of tumor sections and quantification of Ki67-positive cells in the indicated groups (*n* = 6), *** *p* < 0.001. Data are presented as mean ± SD. Statistical significance is indicated in the panels.

To determine whether ZIP7-driven metabolic rewiring translates into malignant phenotypes, we assessed proliferation and apoptotic cell death after ZIP7 silencing. Knockdown of ZIP7 markedly reduced EdU incorporation and colony formation in both PC9 and A549 cells while increasing apoptotic cell death (Figure 3A–E). In vivo, ZIP7-depleted LLC cells formed substantially smaller subcutaneous tumors, with reduced tumor volumes, lower final tumor weights, and decreased Ki67 staining (Figure 3F–I). However, gain-of-function experiments showed that ZIP7 overexpression promoted cell growth, clonogenicity, and tumor progression in H1975 and LLC models (Figure S3A–J). Moreover, ZIP7 knockdown suppressed migration, invasion, and lung metastasis, whereas ZIP7 overexpression exerted the opposite effects (Figure S4A–G). These data establish ZIP7 as a driver of LUAD growth and metastasis.

### 3.4. ZIP7 Restrains ROS Accumulation and Sustains NRF2-Dependent AMPK-mTOR Signaling

**Figure 4:**
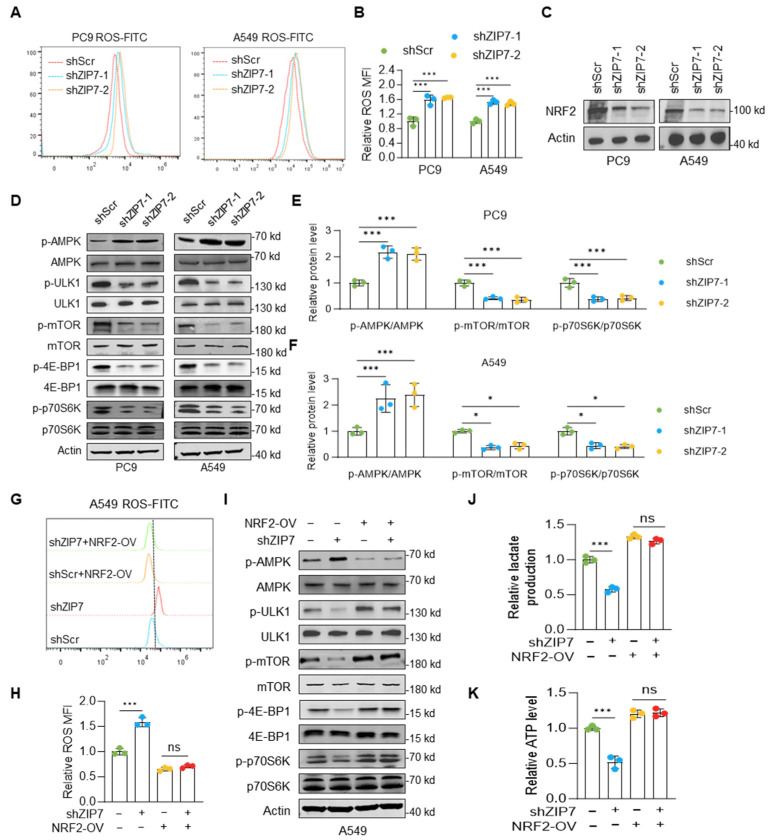
ZIP7 restrains ROS accumulation and maintains NRF2-dependent AMPK-mTOR signaling. (**A**) Flow cytometric analysis of intracellular ROS levels in control and ZIP7-knockdown PC9 and A549 cells. (**B**) Quantification of relative ROS mean fluorescence intensity (MFI) shown in (**A**), *n* = 3, *** *p* < 0.001. (**C**) Immunoblot analysis of NRF2 protein levels in control and ZIP7-knockdown PC9 and A549 cells. Actin served as a loading control. (**D**) Immunoblot analysis of AMPK-mTOR pathway components in control and ZIP7-knockdown PC9 and A549 cells. Actin served as a loading control. (**E**,**F**) Quantification of p-AMPK/AMPK, p-mTOR/mTOR, and p-p70S6K/p70S6K ratios in PC9 and A549 cells shown in (**D**), *n* = 3, * *p* < 0.05, *** *p* < 0.001. (**G**) Flow cytometric analysis of ROS levels in A549 cells with ZIP7 knockdown and NRF2 overexpression. (**H**) Quantification of relative ROS MFI shown in (**G**), *n* = 3, *ns*, no significance, *** *p* < 0.001. (**I**) Immunoblot analysis of AMPK-mTOR pathway proteins in A549 cells with ZIP7 knockdown and NRF2 overexpression. Actin served as a loading control. (**J**) Relative lactate production in A549 cells with ZIP7 knockdown and NRF2 overexpression (*n* = 3), *ns*, no significance, *** *p* < 0.001. (**K**) Relative ATP levels in A549 cells with ZIP7 knockdown and NRF2 overexpression (*n* = 3), *ns*, no significance, *** *p* < 0.001. Data are presented as mean ± SD. Statistical significance is indicated in the panels; *ns*, not significant.

Because redox control is tightly coupled to glycolytic maintenance, we next examined whether ZIP7 regulates intracellular oxidative stress. Flow-cytometric analysis revealed that ZIP7 knockdown increased ROS accumulation in both PC9 and A549 cells (Figure 4A,B), and MitoSOX staining further confirmed increased mitochondrial ROS after ZIP7 depletion (Figure S5A,B). Given the central role of NRF2 in antioxidant defense, we examined NRF2 expression. Concomitantly, NRF2 protein abundance was reduced in ZIP7-silenced cells (Figure 4C), whereas NRF2 mRNA levels remained unchanged (Figure S5C), suggesting that ZIP7 regulates NRF2 predominantly at the post-transcriptional level. Consistently, ZIP7 overexpression increased NRF2 protein in H1975 cells, and NRF2 target genes, including NQO1 and HO1, were downregulated upon ZIP7 knockdown (Figure S5D,E). At the signaling level, ZIP7 depletion increased AMPK activation while suppressing mTOR pathway output, as reflected by increased p-AMPK and reduced phosphorylation of mTOR, p70S6K, and 4E-BP1 (Figure 4D–F). Reconstitution of NRF2 in ZIP7-deficient cells reduced ROS accumulation (Figure 4G,H), restored AMPK-mTOR signaling (Figure 4I), and rescued lactate production and ATP levels (Figure 4J,K). NRF2 overexpression also partially reversed the proliferation and migration defects caused by ZIP7 loss (Figure S5F–H). These findings indicate that ZIP7 maintains glycolytic and malignant phenotypes through an NRF2-dependent redox program that restrains AMPK activation and sustains mTOR signaling.

### 3.5. ZIP7 Stabilizes NRF2 Through Inhibition of GSK3β-Dependent Ubiquitination

**Figure 5:**
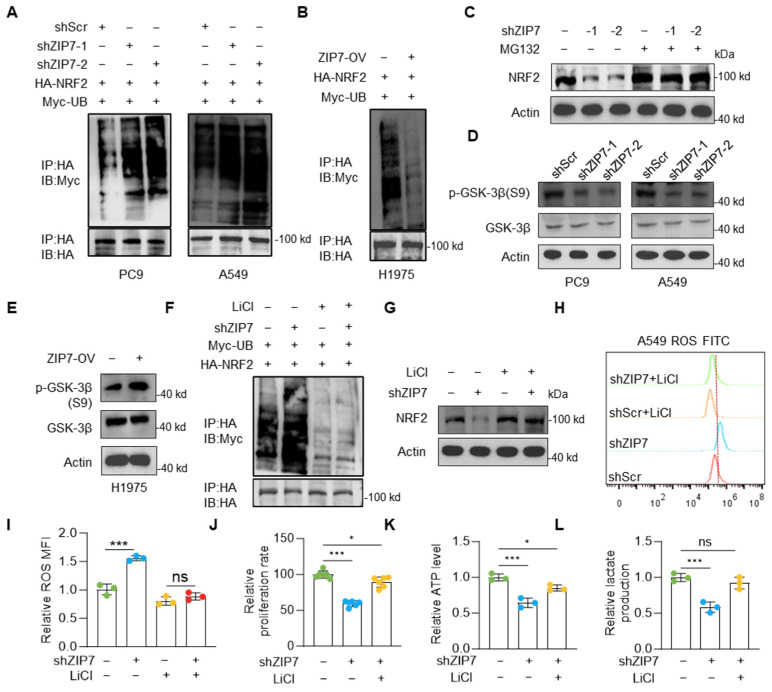
ZIP7 stabilizes NRF2 by limiting GSK3β-dependent ubiquitination. (**A**) Ubiquitination assay showing NRF2 ubiquitination in PC9 and A549 cells with ZIP7 knockdown. Cell lysates were subjected to immunoprecipitation (IP) with anti-HA followed by immunoblotting (IB) with the indicated antibodies. (**B**) Ubiquitination assay showing NRF2 ubiquitination in H1975 cells with or without ZIP7 overexpression. (**C**) Immunoblot analysis of NRF2 protein levels in ZIP7-knockdown cells treated with or without MG132. Actin served as a loading control. (**D**) Immunoblot analysis of p-GSK3β (Ser9) and total GSK3β in control and ZIP7-knockdown PC9 and A549 cells. (**E**) Immunoblot analysis of p-GSK3β (Ser9) and total GSK3β in H1975 cells with or without ZIP7 overexpression. (**F**) Ubiquitination assay showing NRF2 ubiquitination in the presence or absence of LiCl in ZIP7-deficient cells. (**G**) Immunoblot analysis of NRF2 protein levels in ZIP7-deficient cells treated with or without LiCl. Actin served as a loading control. (**H**) Flow cytometric analysis of ROS levels in A549 cells under the indicated conditions. (**I**) Quantification of relative ROS MFI shown in (**H**), *n* = 3, *ns*, no significance, *** *p* < 0.001. (**J**) Relative proliferative rate under the indicated conditions (*n* = 6), * *p* < 0.05, *** *p* < 0.001. (**K**) Relative ATP levels under the indicated conditions (*n* = 3), * *p* < 0.05, *** *p* < 0.001. (**L**) Relative lactate production under the indicated conditions (*n* = 3), *ns*, no significance, *** *p* < 0.001. Data are presented as mean ± SD. Statistical significance is indicated in the panels; *ns*, not significant.

Given that ZIP7 altered NRF2 protein but not NRF2 mRNA, we next investigated whether ZIP7 affects NRF2 stability. Ubiquitination assays showed that ZIP7 knockdown increased NRF2 ubiquitination in PC9 and A549 cells, whereas ZIP7 overexpression decreased NRF2 ubiquitination in H1975 cells (Figure 5A,B). Moreover, proteasome inhibition with MG132 restored NRF2 protein levels in ZIP7-deficient cells (Figure 5C), supporting a proteasome-dependent mechanism of NRF2 loss. Because GSK3β is a well-established negative regulator of NRF2 stability, we examined this axis and found that ZIP7 knockdown reduced inhibitory phosphorylation of GSK3β at Ser9 in PC9 and A549 cells, whereas ZIP7 overexpression increased p-GSK3β (Ser9) in H1975 cells (Figure 5D,E). Treatment with lithium chloride (LiCl, 10 mM), a well-established inhibitor of GSK-3β activity, reduced NRF2 ubiquitination and restored NRF2 protein abundance in ZIP7-deficient cells (Figure 5F,G). LiCl also lowered ROS levels and rescued proliferation, ATP production, and lactate output (Figure 5H–L). Together, these data indicate that ZIP7 stabilizes NRF2 by promoting inhibitory phosphorylation of GSK3β and thereby limiting GSK3β-dependent NRF2 degradation.

### 3.6. ZIP7-Driven Lactate Accumulation Programs M2-like Macrophage Polarization and PD-L1 Expression

**Figure 6:**
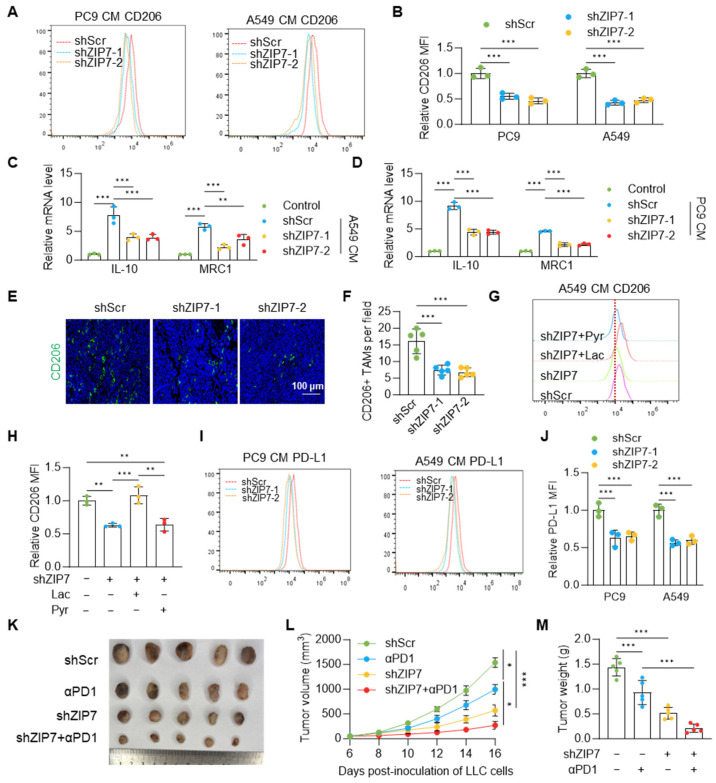
ZIP7-driven lactate production promotes macrophage M2-like polarization and enhances the response to anti-PD-1 therapy. (**A**) Flow cytometric analysis of CD206 expression in macrophages treated with conditioned medium (CM) from control or ZIP7-knockdown PC9 and A549 cells. (**B**) Quantification of relative CD206 MFI shown in (**A**), *n* = 3, *** *p* < 0.001. (**C**,**D**) Relative mRNA expression of the M2-associated markers IL-10 and MRC1 in macrophages treated with CM from A549 cells (**C**) or PC9 cells (**D**) under the indicated conditions (*n* = 3), ** *p* < 0.01, *** *p* < 0.001. (**E**) Representative immunofluorescence staining of CD206-positive tumor-associated macrophages (TAMs) in tumor sections from the indicated groups. Scale bar, 100 μm. (**F**) Quantification of CD206-positive TAMs per field shown in (**E**), *n* = 5, *** *p* < 0.001. (**G**) Flow cytometric analysis of CD206 expression in macrophages treated with conditioned medium from A549 cells, with lactate or pyruvate supplementation as indicated. (**H**) Quantification of relative CD206 MFI shown in (**G**), *n* = 3, ** *p* < 0.01, *** *p* < 0.001. (**I**) Flow cytometric analysis of PD-L1 expression in macrophages treated with CM from control or ZIP7-knockdown PC9 and A549 cells. (**J**) Quantification of relative PD-L1 MFI shown in (**I**), *n* = 3, *** *p* < 0.001. (**K**) Representative images of tumors from LLC-bearing mice treated with shZIP7 and/or anti-PD-1. (**L**) Tumor growth curves of the indicated treatment groups (*n* = 5), * *p* < 0.05, *** *p* < 0.001. (**M**) Final tumor weights of the indicated groups (*n* = 5), *** *p* < 0.001. Data are presented as mean ± SD. Statistical significance is indicated in the panels.

Because lactate is a major immunomodulatory output of tumor glycolysis, we next asked whether ZIP7 shapes macrophage polarization. Conditioned medium (CM) from ZIP7-knockdown PC9 or A549 cells significantly reduced CD206 MFI (Figure 6A,B) and decreased the expression of the M2-associated markers IL-10 and MRC1 (Figure 6C,D) when compared to the control. In vivo, tumors derived from ZIP7-deficient LLC cells contained fewer CD206-positive TAMs (Figure 6E,F). Importantly, supplementation with exogenous lactate, but not pyruvate, restored the reduction in CD206 caused by ZIP7 knockdown (Figure 6G,H), indicating that lactate acts as a key downstream mediator for ZIP7-dependent macrophage M2 polarization. As M2 macrophages are known to upregulate the immune checkpoint PD-L1, we next examined its expression. In parallel, macrophages exposed to CM from ZIP7-silenced LUAD cells exhibited reduced PD-L1 expression (Figure 6I,J). This finding suggested that targeting ZIP7 could potentiate anti-PD-1 immunotherapy by alleviating PD-L1-mediated immunosuppression. Functionally, genetic ZIP7 inhibition enhanced the antitumor efficacy of anti-PD-1 therapy in vivo, with the combination group showing the smallest tumors, the slowest growth, and the lowest tumor weights (Figure 6K–M). These results support a non-cell-autonomous role for ZIP7 in coupling tumor glycolysis to an immunosuppressive macrophage phenotype.

### 3.7. Pharmacologic Inhibition of ZIP7 by ZP74 Suppresses LUAD Progression and Enhances Immunotherapy Efficacy

**Figure 7:**
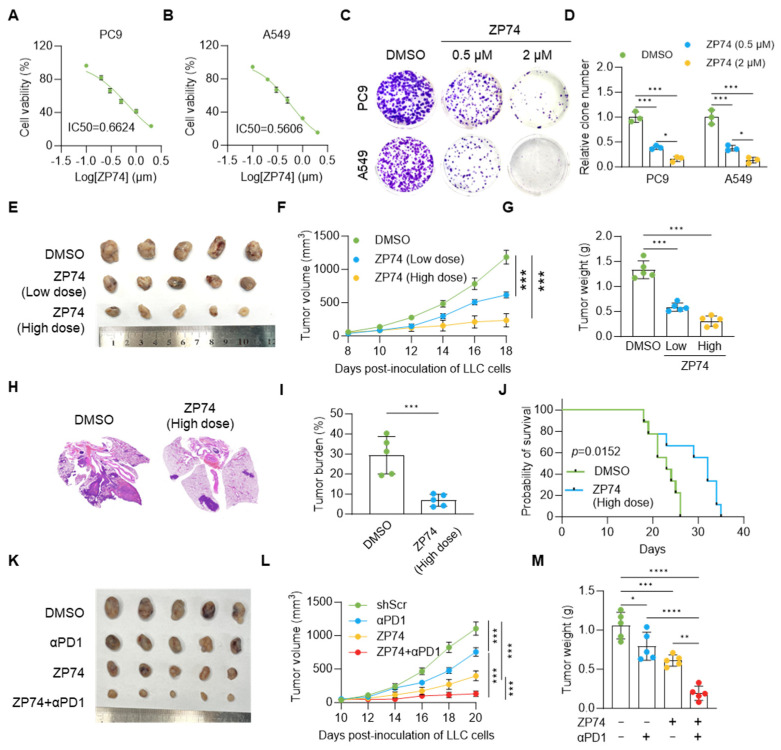
Pharmacological inhibition of ZIP7 by ZP74 suppresses LUAD progression and potentiates anti-PD-1 therapy. (**A**,**B**) Cell viability curves and IC50 values of ZP74 in PC9 and A549 cells. (**C**) Representative colony formation images of PC9 and A549 cells treated with DMSO or ZP74 at the indicated concentrations. (**D**) Quantification of relative colony numbers shown in (**C**), *n* = 3, * *p* < 0.05, *** *p* < 0.001. (**E**) Representative images of subcutaneous tumors from LLC-bearing mice treated with DMSO or ZP74 at low or high doses. (**F**) Tumor growth curves of the indicated treatment groups (*n* = 5), *** *p* < 0.001. (**G**) Final tumor weights of the indicated treatment groups (*n* = 5), *** *p* < 0.001. (**H**) Representative hematoxylin and eosin staining of lung sections from the orthotopic lung tumor model treated with DMSO or high-dose ZP74. (**I**) Quantification of tumor burden in the orthotopic lung tumor model (*n* = 5), *** *p* < 0.001. (**J**) Kaplan–Meier survival analysis of mice in the orthotopic lung tumor model treated with DMSO or high-dose ZP74 (*n* = 9). (**K**) Representative images of tumors from LLC-bearing mice treated with DMSO, anti-PD-1, ZP74, or the combination of ZP74 and anti-PD-1. (**L**) Tumor growth curves of the indicated treatment groups (*n* = 5), *** *p* < 0.001. (**M**) Final tumor weights of the indicated treatment groups (*n* = 5), * *p* < 0.05, ** *p* < 0.01, *** *p* < 0.001, **** *p* < 0.0001. Data are presented as mean ± SD unless otherwise indicated. Statistical significance is indicated in the panels.

Finally, we evaluated whether pharmacologic ZIP7 inhibition by ZP74 (also known as NVS-ZP7-4), a reported chemical inhibitor of ZIP7, phenocopies the genetic effects observed above. ZP74 reduced the viability of LUAD cells in a dose-dependent manner, with submicromolar IC50 values in both cell lines (Figure 7A,B), and markedly suppressed colony formation, EdU incorporation, and induced apoptosis, with no evidence of off-target effects (Figure 7C,D and Figure S6A–D). In vivo, ZP74 treatment inhibited LLC subcutaneous tumor growth and reduced final tumor weights in a dose-dependent manner (Figure 7E–G). In an orthotopic lung tumor model, high-dose ZP74 decreased tumor burden and significantly prolonged survival (Figure 7H–J). Moreover, ZP74 enhanced the response to anti-PD-1 therapy, with the combination treatment achieving the strongest suppression of tumor growth and tumor weight (Figure 7K–M). Further analyses showed that ZP74 inhibited migration, invasion, wound healing, and lung metastasis while exhibiting minimal toxicity toward BEAS-2B cells and no obvious adverse effects on body weight or major organs (Figures S7A–I and S8A–C). Collectively, these data identify ZIP7 as a therapeutically actionable target in LUAD.

## 4. Discussion

This study identifies ZIP7 as a central coordinator of LUAD progression by functionally linking zinc flux to redox buffering, glycolytic reinforcement, and immunosuppressive microenvironmental remodeling. Using an siRNA screen across the ZIP transporter family, coupled with patient dataset integration and mechanistic dissection, we demonstrate that ZIP7 is both clinically enriched in LUAD and mechanistically required for robust glycolytic output and malignant fitness. These findings align with the concept that aerobic glycolysis is a selected trait in cancers and can be stabilized by upstream signaling and redox programs [23].

A key contribution of our work is positioning ZIP7 upstream of an NRF2-dependent ROS checkpoint that modulates AMPK–mTOR signaling. As a pivotal regulator of glycolysis, mTOR promotes this metabolic process when active; conversely, its inhibition exerts multifaceted suppression of glycolysis through downregulation of key factors including HIF-1α, glucose transporters, and glycolysis enzymes, thereby directly restraining tumor malignant progression [24]. AMPK, as a key negative regulator of mTOR, can be activated by cell stress signals such as ROS [25]. Upon activation, AMPK inhibits mTOR by phosphorylating Raptor or activating TSC2 [26]. Consistent with this regulatory axis, our study confirms that ZIP7 deficiency upregulates ROS, which in turn activates AMPK to inhibit mTOR signaling, ultimately suppressing tumor glycolysis, growth, and metastasis.

ZIP7, an ER-resident zinc transporter, has been shown in previous studies to induce ER stress when dysregulated [27]. As the primary intracellular Ca^2+^ reservoir, ER stress not only forms a bidirectional positive feedback loop with ROS but also further exacerbates oxidative stress via aberrant calcium release from ER stores [28,29,30]. This suggests a potential intricate crosstalk between ZIP7, ER stress, ROS and calcium signaling. Future in-depth investigation of this crosstalk holds promise for developing more precise and clinically translatable anticancer intervention strategies.

NRF2 serves as a central regulator of cellular redox homeostasis. Under basal conditions, NRF2 is maintained at low levels through Keap1–Cul3-mediated ubiquitination and proteasomal degradation [31,32,33]. In addition, a Keap1-independent route exists in which GSK3β creates a phosphodegron in NRF2 that is recognized by β-TrCP, targeting NRF2 for degradation via the SCF ubiquitin ligase complex [34,35]. GSK3β can also act upstream of Fyn kinase to facilitate NRF2 nuclear export and degradation [36]. Our data support a model in which ZIP7 increases inhibitory phosphorylation of GSK3β, thereby limiting GSK3β-dependent NRF2 turnover, reducing NRF2 ubiquitination, and sustaining NRF2 protein stability. This is conceptually consistent with prior work showing that intracellular zinc transport can activate proliferative signaling and kinase cascades downstream of ZIP7 [37], and with broader evidence that zinc influx can impinge on GSK3β activity in EMT contexts [38]. Building on ZIP7’s role in ER zinc release, we posit that its loss reduces cytosolic zinc, potentially triggering GSK3β dephosphorylation/activation—either through direct allosteric modulation or via zinc-sensitive signaling nodes. Future studies will definitively map this upstream signaling by integrating ICP-MS, zinc reconstitution, and in vitro kinase assays.

Beyond tumor-intrinsic rewiring, our work highlights a tumor–immune coupling mechanism mediated by lactate [39]. Lactate is not merely a metabolic byproduct; it can act as an immunomodulatory metabolite that promotes macrophage M2-like polarization and pro-tumor functions [40]. We demonstrate that ZIP7-driven glycolysis elevates lactate and is required for macrophage M2 polarization in co-culture systems, and that exogenous lactate rescues impaired M2 polarization caused by ZIP7 depletion. Notably, studies in oxamic acid-pretreated breast cancer cells showed that lactate, but not HCl, restores M2 polarization, confirming lactate-specific effects independent of pH [41]. Our data further demonstrate that ZIP7-mediated macrophage polarization depends on lactate rather than acidic pH. M2 macrophages drive tumor progression through mechanisms such as promoting angiogenesis and fostering an immunosuppressive TME, establishing them as compelling therapeutic targets [42]. Current clinical approaches mainly involve blocking TAMs recruitment or reprogramming M2 macrophages to M1-like phenotypes. Nevertheless, the clinical success of these single-target strategies has been limited [43]. In contrast, ZIP7 inhibition acts dually to suppress tumor cell proliferation and lactate-driven M2 macrophage polarization, thereby representing a promising multi-target therapeutic strategy.

Our data also provide translational rationale. Pharmacologic inhibition of ZIP7 with ZP74 (also known as NVS-ZP7-4), a reported chemical tool compound targeting ZIP7-mediated ER zinc release, phenocopies genetic ZIP7 depletion, suppresses tumor growth and metastatic dissemination, and shows limited systemic toxicity in our models. This selective toxicity arises, at least in part, likely due to the fact that tumor cells tend to be highly dependent on ZIP7 for zinc homeostasis and survival signaling, whereas normal cells typically possess functional redundancy and stronger stress compensatory capacity. Furthermore, we found that CM from ZIP7-knockdown LUAD cells significantly downregulated PD-L1 expression in macrophages. This observation aligns with lactate’s established role in promoting PD-L1 expression [44,45]. While PD-1/PD-L1 inhibitors have revolutionized LUAD treatment, their efficacy is limited by low response rates and drug resistance [46]. Here, we demonstrated that ZIP7 inhibition, whether achieved through genetic depletion or pharmacological intervention with ZP74 exerts a synergistic effect with anti-PD-1 therapy. These findings establish ZIP7 inhibition as a versatile approach with dual benefits in mono- and combination therapy. Moving forward, single-cell RNA sequencing will dissect immune cell population dynamics post-ZIP7 intervention to further clarify the intricate immunoregulatory mechanisms mediated by ZIP7.

## 5. Conclusions

Taken together, our work identifies ZIP7 as a pivotal orchestrator of LUAD pathogenesis, integrating AMPK/mTOR-driven glycolysis with lactate-mediated immunosuppression. ZIP7 inhibition not only directly suppresses tumor growth but also sensitizes LUAD to anti-PD-1 therapy. Thus, ZIP7 is a promising therapeutic target for LUAD with the potential to overcome current therapy limitations.

## Data Availability

No datasets were generated during the current study. The data supporting this study’s findings are available from the corresponding authors upon reasonable request.

## Supplementary Materials

The following supporting information can be downloaded at: https://www.mdpi.com/article/10.3390/biomedicines14061262/s1, Figure S1. ZIP7 is prioritized from the ZIP family screen and is associated with glycolytic features in LUAD. Figure S2. ZIP7 overexpression enhances glycolysis in H1975 cells. Figure S3. ZIP7 overexpression promotes LUAD cell proliferation and tumor growth. Figure S4. ZIP7 regulates migration, invasion, and metastatic colonization of LUAD cells. Figure S5. ZIP7 regulates mitochondrial ROS, NRF2 target gene expression, and NRF2-mediated rescue phenotypes. Figure S6. ZP74 suppresses proliferation and induces apoptosis in LUAD cells. Figure S7. ZP74 inhibits migration, invasion, wound healing, and lung metastasis of LUAD cells. Figure S8. ZP74 exhibits limited toxicity in vitro and in vivo. Table S1. shRNA targeted sequences. Table S2. Antibodies. Table S3. related to Methods. RT-PCR primers.

## Abbreviations

AMPK	AMP-activated protein kinase
ATP	Adenosine triphosphate
CCK-8	Cell Counting Kit-8
CM	Conditioned medium
DAB	3,3′-Diaminobenzidine
DAPI	4′,6-Diamidino-2-phenylindole
DCFH-DA	2′,7′-Dichlorodihydrofluorescein diacetate
ECAR	Extracellular acidification rate
ECL	Enhanced chemiluminescence
EdU	5-Ethynyl-2′-deoxyuridine
ER	Endoplasmic reticulum
FBS	Fetal bovine serum
GSK3β	Glycogen synthase kinase 3 beta
HO1	Heme oxygenase 1
HRP	Horseradish peroxidase
IB	Immunoblotting
IF	Immunofluorescence
IHC	Immunohistochemistry
IL-10	Interleukin-10
IP	Immunoprecipitation
LUAD	Lung adenocarcinoma
MFI	Mean fluorescence intensity
MRC1	Mannose receptor C-type 1
mTOR	Mechanistic target of rapamycin
NRF2	Nuclear factor erythroid 2-related factor 2
NQO1	NAD(P)H quinone dehydrogenase 1
PBS	Phosphate-buffered saline
PD-1	Programmed cell death protein 1
PD-L1	Programmed death-ligand 1
PI	Propidium iodide
PVDF	Polyvinylidene fluoride
qPCR	Quantitative polymerase chain reaction
ROS	Reactive oxygen species
RPMI	Roswell Park Memorial Institute medium
SDS-PAGE	Sodium dodecyl sulfate-polyacrylamide gel electrophoresis
siRNA	Small interfering RNA
TAMs	Tumor-associated macrophages
WB	Western blot
ZIP7	Zinc transporter 7 (SLC39A7)
