# Supplementary material for: ZIP7 Drives Glycolytic Reprogramming and Lactate-Mediated Immune Remodeling in Lung Adenocarcinoma Through GSK3β-NRF2 Signaling
Source: Biomedicines. 2026 Jun 1;14(6):1262. doi: 10.3390/biomedicines14061262 (PMC13296730; doi:10.3390/biomedicines14061262)
Supplement: Supplementary file 1 [file biomedicines-14-01262-s001.zip › biomedicines-4260077-supplementary.pdf]

Supplemental information

ZIP7 Drives Glycolytic Reprogramming and Lactate-Mediated Immune Remodelling in Lung Adenocarcinoma Through GSK3β-NRF2 Signaling

The file includes:

1. **Supplemental Figures S1 to S8**
2. **Supplemental Tables S1 to S3**

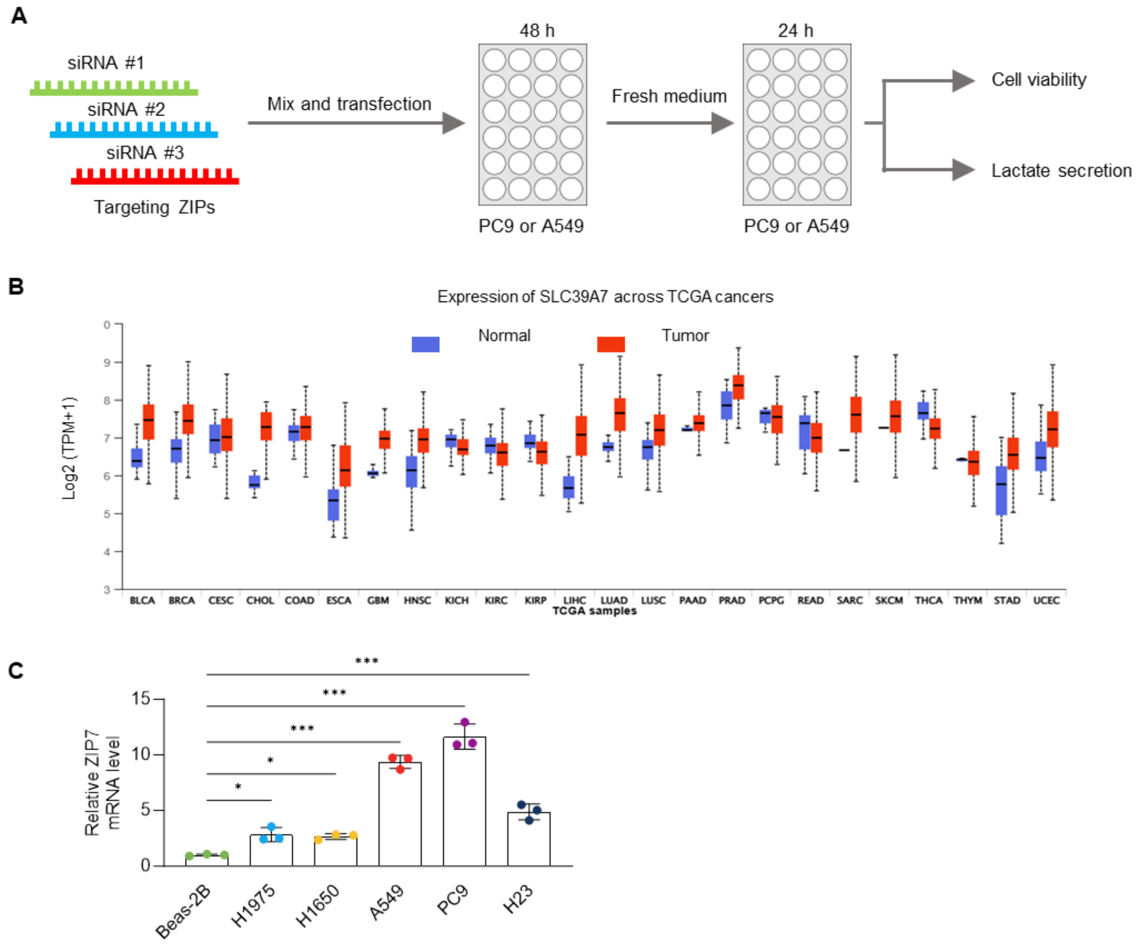

Figure S1. ZIP7 is prioritized from the ZIP family screen and is associated with glycolytic features in LUAD.

(A) Schematic illustration of the screening workflow used to assess the effects of individual ZIP family members on cell viability and lactate secretion in PC9 and A549 cells following transient siRNA-mediated silencing.

(B) Analysis of SLC39A7 (ZIP7) expression in pan-cancer by UALCAN

(https://ualcan.path.uab.edu/analysis.html).

(C) q-PCR analysis of ZIP7 mRNA expression in the indicated lung cell lines.

Data are presented as mean ± SD unless otherwise indicated. Statistical significance is indicated in the panels.

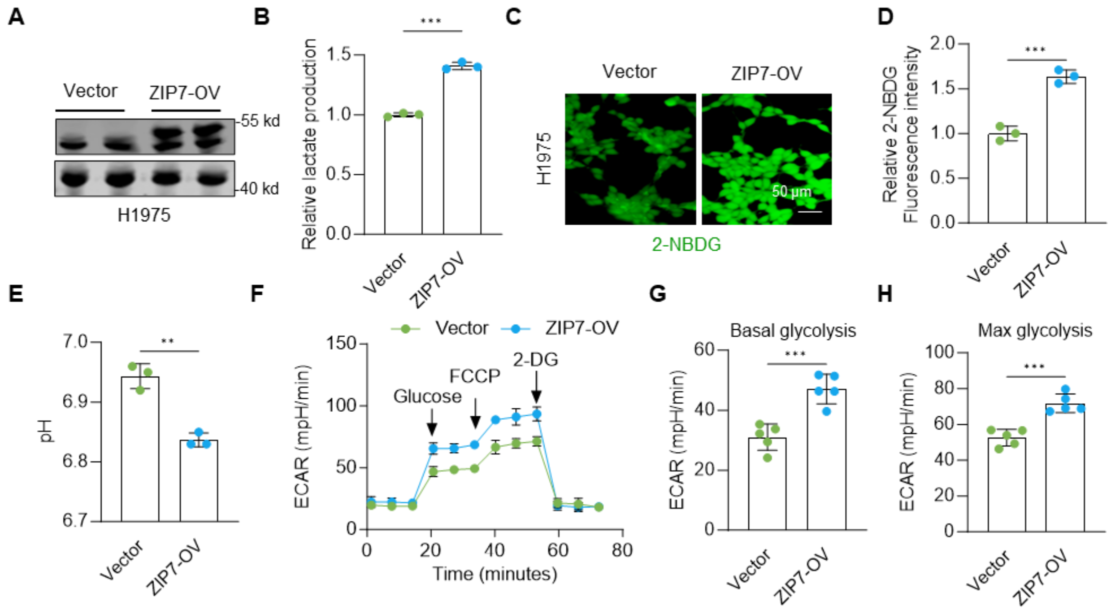

Figure S2. ZIP7 overexpression enhances glycolysis in H1975 cells.

(A) Western blot analysis confirms ZIP7 overexpression (ZIP7-OV) in H1975 cells, with β-actin as loading control.

(B) Relative lactate production in H1975 cells transfected with vector or ZIP7-overexpressing constructs.

(C) Representative images of 2-NBDG uptake in vector- and ZIP7-overexpressing H1975 cells. Scale bar, 50 μm.

(D) Quantification of relative 2-NBDG fluorescence intensity shown in (C).

(E) Culture medium pH was measured in vector- and ZIP7-overexpressing H1975 cells.

(F) Extracellular acidification rate (ECAR) profiles of vector- and ZIP7-overexpressing H1975 cells during Seahorse glycolysis stress testing.

(G, H) Quantification of basal glycolysis and maximal glycolysis derived from the ECAR assays shown in (F).

Data are presented as mean ± SD. Statistical significance is indicated in the panels.

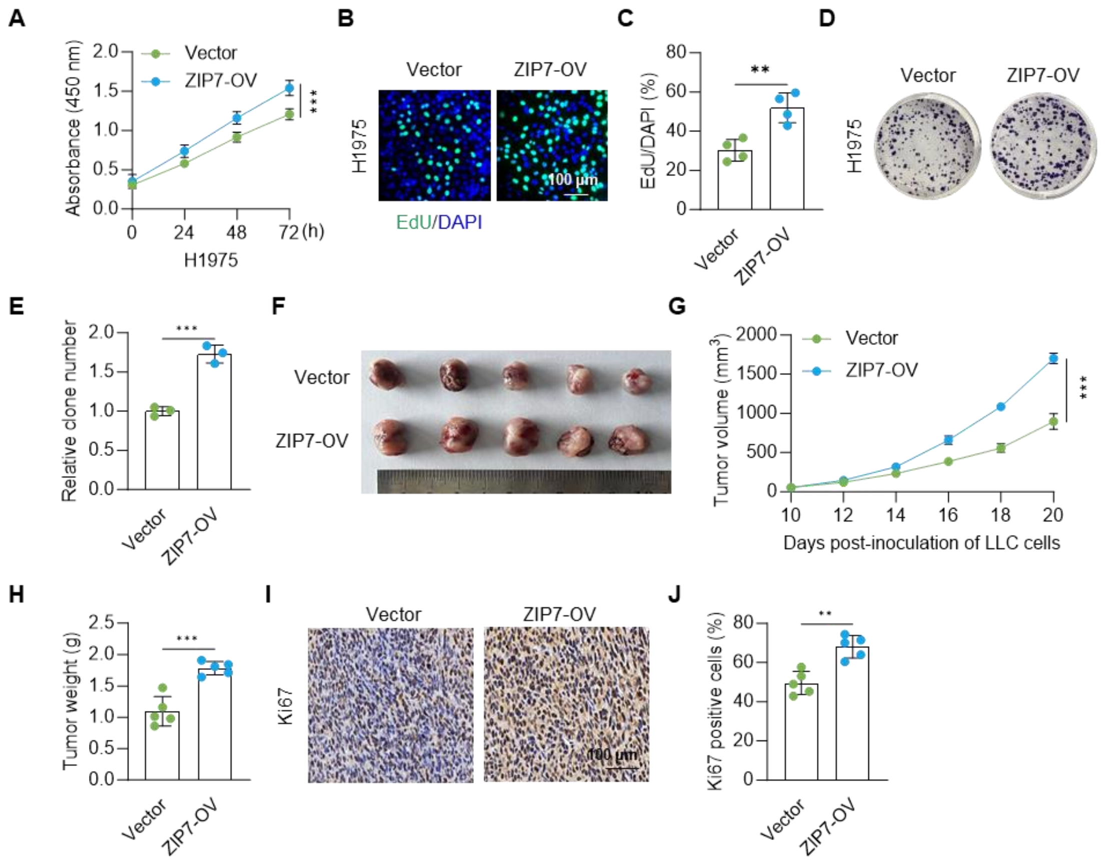

Figure S3. ZIP7 overexpression promotes LUAD cell proliferation and tumor growth.

(A) CCK8 analysis of cell proliferation in H1975 cells transfected with vector or ZIP7-overexpressing constructs.

(B) Representative EdU staining images of vector- and ZIP7-overexpressing H1975 cells. Scale bar, 100 μm.

(C) Quantification of EdU-positive cells shown in (B).

(D). Representative colony formation images of vector- and ZIP7-overexpressing H1975 cells.

(E) Quantification of colony numbers shown in (D).

(F) Representative images of subcutaneous tumors derived from LLC cells expressing vector or ZIP7-overexpressing constructs.

(G) Tumor growth curves of the indicated groups in the LLC subcutaneous xenograft model.

(H) Final tumor weights from the indicated groups.

(I) Representative Ki67 immunohistochemical staining of tumor sections from the

indicated groups. Scale bar, 100 μm.

(J) Quantification of Ki67-positive cells shown in (I).

Data are presented as mean ± SD. Statistical significance is indicated in the panels.

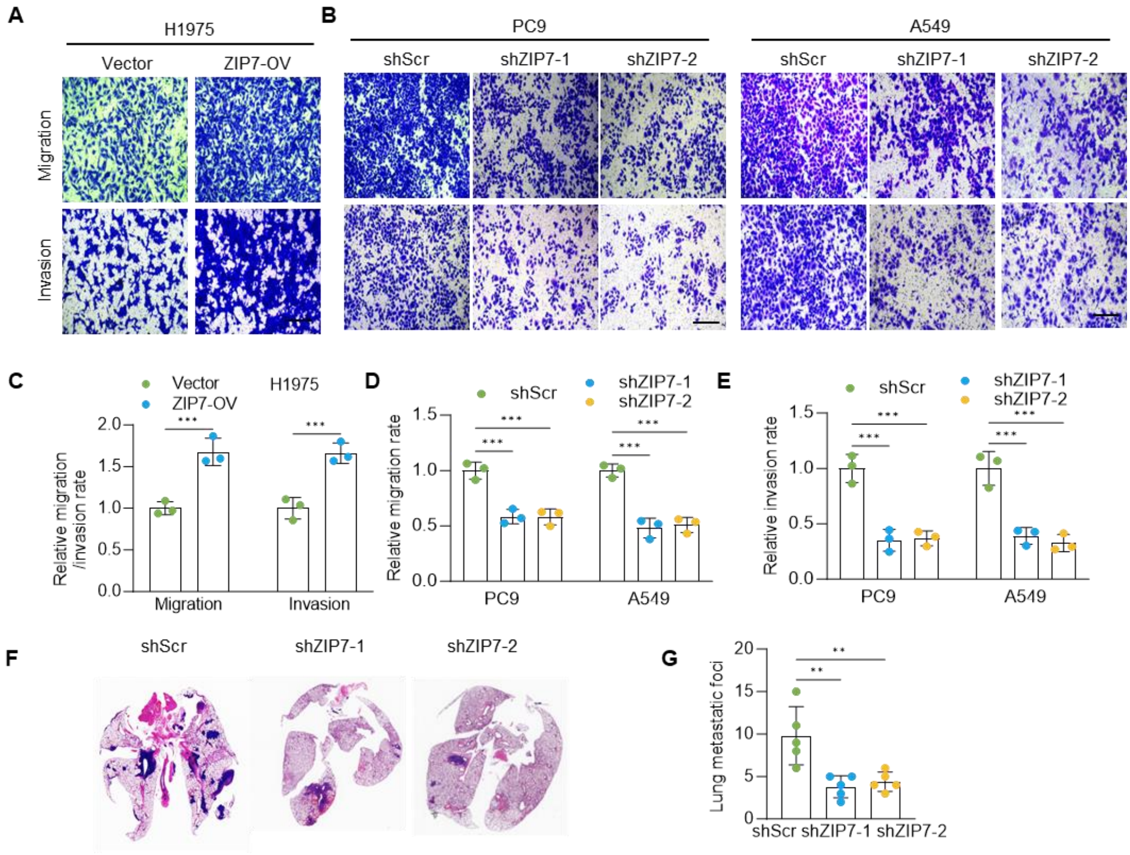

Figure S4. ZIP7 regulates migration, invasion, and metastatic colonization of LUAD cells.

(A) Representative transwell migration and invasion images of H1975 cells transfected with vector or ZIP7-overexpressing constructs. Scale bar, 100 μm.

(B) Representative transwell migration and invasion images of control and ZIP7-knockdown PC9 and A549 cells. Scale bar, 100 μm.

(C) Quantification of relative migration and invasion rates shown in (A).

(D, E) Quantification of relative migration and invasion rates shown in (B).

(F) Representative images of lung metastatic nodules in the indicated groups.

(G) Quantification of lung metastatic foci shown in (F).

Data are presented as mean ± SD. Statistical significance is indicated in the panels.

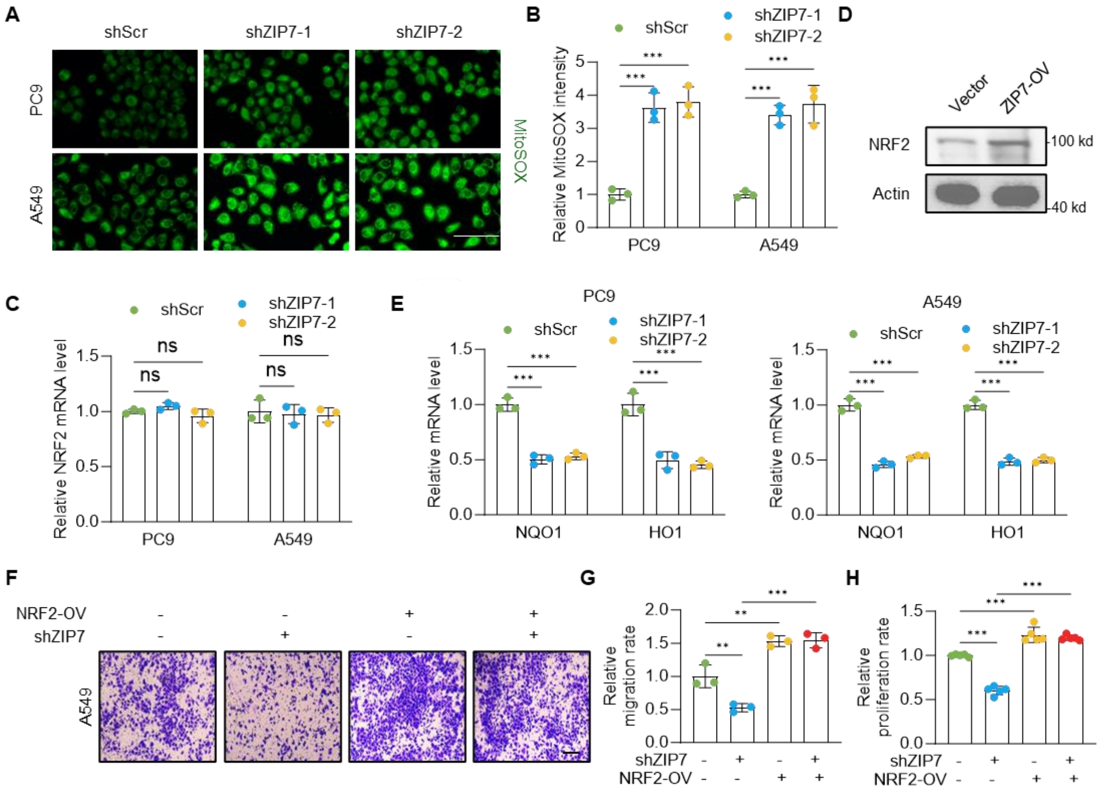

Figure S5. ZIP7 regulates mitochondrial ROS, NRF2 target gene expression, and NRF2-mediated rescue phenotypes.

(A) Representative MitoSOX staining images of control and ZIP7-knockdown PC9 and A549 cells. Scale bar, 100 μm.

(B) Quantification of relative MitoSOX fluorescence intensity shown in (A).

(C) Relative NRF2 mRNA expression in control and ZIP7-knockdown PC9 and A549 cells.

(D). Western blot analysis showing NRF2 levels in control and ZIP7 overexpression H1975 cells.

(E) Relative mRNA expression of NRF2 target genes, including NQO1 and HO1, in control and ZIP7-knockdown PC9 and A549 cells.

(F, G) Transwell analysis of cell migration in H1975 cells after transfected with ZIP7 knockdown and NRF2 overexpression lentivirus. The quantification of migration rate was shown as a bar graph. Scale bar, 100 μm.

(H) Relative cell viability in control and ZIP7 knockdown A549 cells treated with or without NRF2 overexpression.

Data are presented as mean ± SD. Statistical significance is indicated in the panels; ns, not significant.

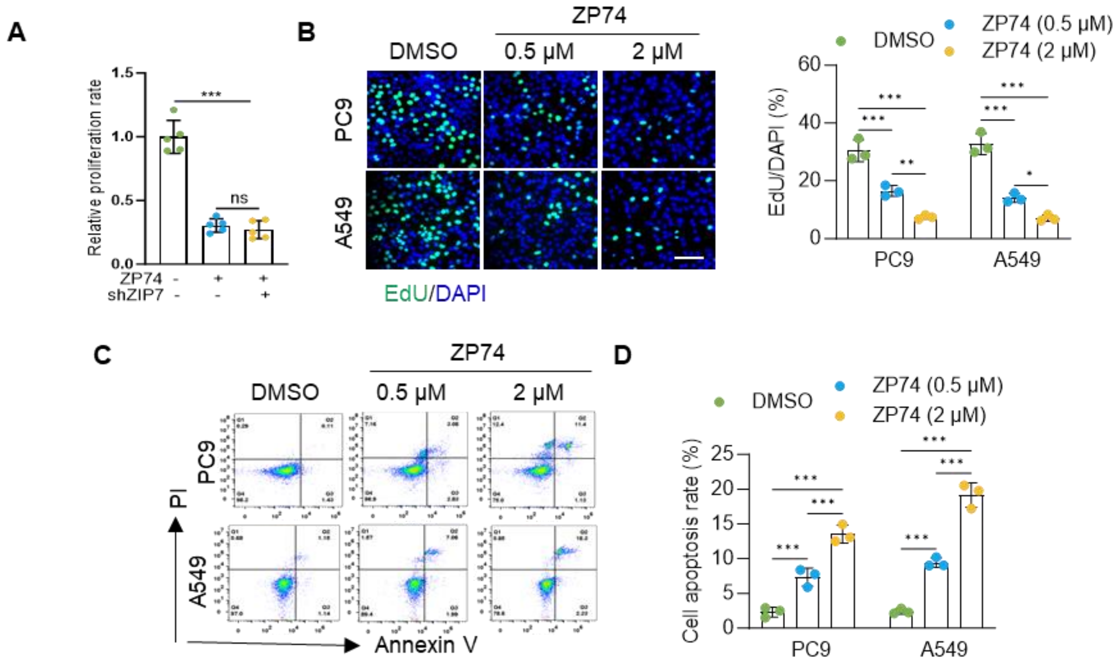

Figure S6. ZP74 suppresses proliferation and induces apoptosis in LUAD cells.

(A) Cell proliferation was evaluated in ZIP7-knockdown and control cells treated with ZP74.

(B) Representative EdU staining images of PC9 and A549 cells treated with DMSO or ZP74 at the indicated concentrations. Scale bar, 100 μm. Quantification of EdU-positive cells shown in (B).

(C) Flow cytometric analysis of apoptosis in PC9 and A549 cells treated with DMSO or ZP74 at the indicated concentrations.

(D) Quantification of apoptotic cell percentages shown in (C).

Data are presented as mean ± SD. Statistical significance is indicated in the panels.

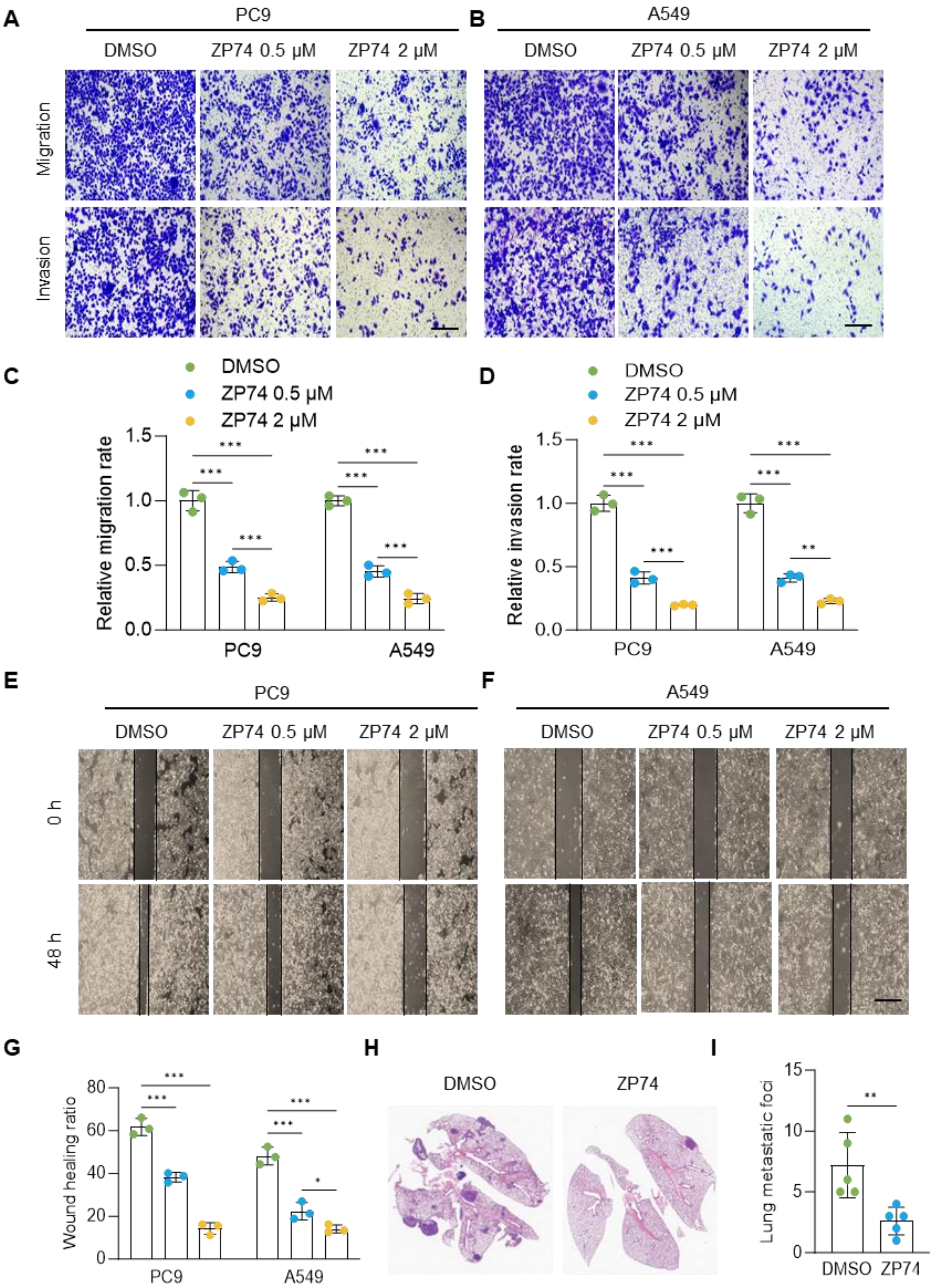

Figure S7. ZP74 inhibits migration, invasion, wound healing, and lung metastasis of LUAD cells.

(A, B) Representative transwell migration and invasion images of PC9 and A549 cells treated with DMSO or ZP74 at the indicated concentrations. Scale bar, 100 μm.

(C, D) Quantification of relative migration and invasion rates shown in (A, B).

(E, F) Representative wound-healing images of PC9 and A549 cells treated with DMSO or ZP74 at the indicated concentrations. Scale bar, 50 μm.

(G) Quantification of wound-healing ratios shown in (E) and (F).

(H) Representative images of lung metastatic nodules in mice treated with DMSO or ZP74.

(I) Quantification of lung metastatic foci shown in (H).

Data are presented as mean ± SD. Statistical significance is indicated in the panels.

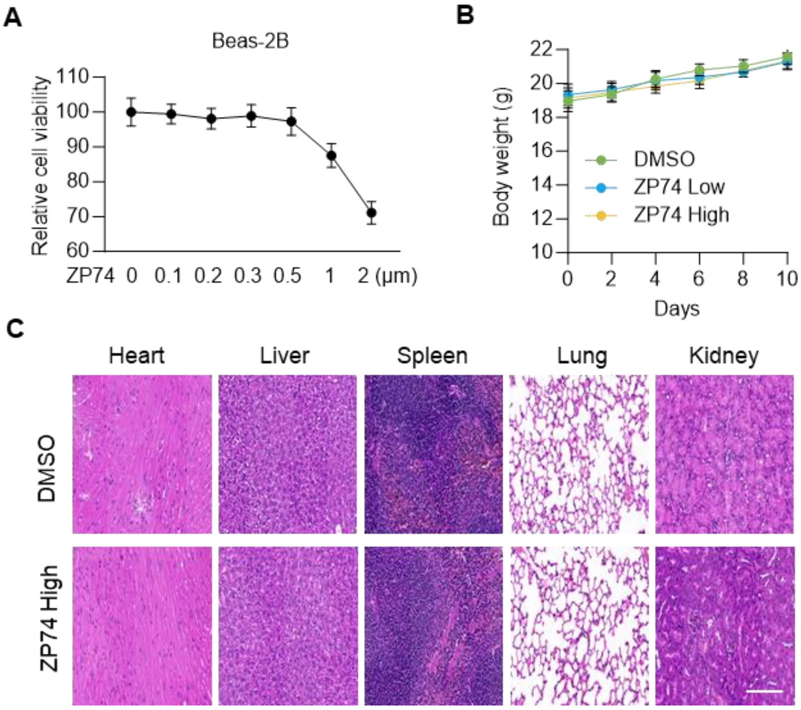

Figure S8. ZP74 exhibits limited toxicity in vitro and in vivo.

(A) Relative viability of BEAS-2B cells treated with the indicated concentrations of ZP74.

(B) Body weight curves of mice treated with DMSO, low-dose ZP74, or high-dose ZP74.

(C) Representative hematoxylin and eosin staining of major organs, including heart, liver, spleen, lung, and kidney, from mice receiving the indicated treatments. Scale bar, 100 μm.

Data are presented as mean ± SD unless otherwise indicated.

Supplementary tables

Supplementary Table S1, shRNA targeted sequences.

Name	Sequence	Function
shZIP7-1# human	GTTGCATTTGGAAGGCTAAAT	shRNA Target
shZIP7-2# human	GCCTTTCTTGTCGTGGAGAAA	shRNA Target
shZIP7-1# mouse	CTCATCCCAGTAGAATCTAAC	shRNA Target
shZIP7-2# mouse	CGGGCACTCACATGAAGATTT	shRNA Target

Supplementary Table S2, Antibodies

Antibody name	Vendor or Source	Catalog No.	Dilution factors
ZIP7	abcam	ab254566	WB: 1:1000<br>IHC: 1:200
Ki67	abcam	ab16667	IHC: 1:200
NRF2	CST	#33649	WB: 1:1000
AMPK	CST	#97117	WB: 1:1000
p-AMPK	abmart	T55608	WB: 1:1000
ULK1	CST	#8054	WB: 1:1000
p-ULK1	CST	#14202	WB: 1:1000
mTOR	CST	#2983	WB: 1:1000
p-mTOR	CST	#5536	WB: 1:1000
4E-BP1	CST	#9644	WB: 1:1000
p-4E-BP1	CST	#2855	WB: 1:1000
p70S6K	CST	#2708	WB: 1:1000
p-p70S6K	CST	#9234	WB: 1:1000
GSK-3β	abmart	TA5016	WB: 1:1000
p-GSK-3β(S9)	abmart	T40070	WB: 1:1000
HA	CST	#3724	WB: 1:2000<br>IP: 1:50
Myc	abmart	M20002	WB: 1:2000
CD206	CST	#24595	IF: 1:100
Actin	abmart	T40104	WB: 1:3000

Supplementary Table S3, related to Methods. RT-PCR primers.

Name	Forward Primer (5' -> 3')	Reverse Primer (5' -> 3')

IL-10	GACTTTAAGGGTTACCTGGGTTG	TCACATGCGCCTTGATGTCTG
MRC-1	TCCGGGTGCTGTTCTCCTA	CCAGTCTGTTTTTGATGGCACT
ZIP7	GGACACGCTCACAGTCATACA	CTCCTCGCCTCTTCTGAACC
NQO1	GAAGAGCACTGATCGTACTGGC	GGATACTGAAAGTTCGCAGGG
HO1	AAGACTGCGTTCCTGCTCAAC	AAAGCCCTACAGCAACTGTCG
